# Outcome and patients' satisfaction after functional treatment of acute lateral ankle injuries at emergency departments versus family doctor offices

**DOI:** 10.1186/1471-2296-9-69

**Published:** 2008-12-23

**Authors:** Patrik R Schwab, Lorin M Benneker, Stefan Eggli, Heinz Zimmermann, Aristomenis K Exadaktylos

**Affiliations:** 1 Department for Orthopaedic Surgery, Inselspital, University of Berne, Berne, Switzerland; 2 Emergency Unit, Inselspital, University of Berne, Berne, Switzerland

## Abstract

**Background:**

In some Western countries, more and more patients seek initial treatment even for minor injuries at emergency units of hospitals. The initial evaluation and treatment as well as aftercare of these patients require large amounts of personnel and logistical resources, which are limited and costly, especially if compared to treatment by a general practitioner. In this study, we investigated whether outsourcing from our level 1 trauma center to a general practitioner has an influence on patient satisfaction and compliance.

**Methods:**

This prospective, randomized study, included n = 100 patients who suffered from a lateral ankle ligament injury grade I-II (16, 17). After radiological exclusion of osseous lesions, the patients received early functional treatment and were shown physical therapy exercises to be done at home, without immobilization or the use of stabilizing ortheses. The patients were randomly assigned into two groups of 50 patients each: Group A (ER): Follow-up and final examination in the hospital's emergency unit. Group B (GP): Follow-up by general practitioner, final examination at hospital's emergency unit. The patients were surveyed regarding their satisfaction with the treatment and outcome of the treatment.

**Results:**

Female and male patients were equally represented in both groups. The age of the patients ranged from 16 – 64 years, with a mean age of 34 years (ER) and 35 years (GP). 98% (n = 98) of all patients were satisfied with their treatment, and 93% (n = 93) were satisfied with the outcome. For these parameters no significant difference between the two groups could be noted (p = 0.7406 and 0.7631 respectively). 39% of all patients acquired stabilizing ortheses like ankle braces (Aircast, Malleoloc etc.) on their own initiative. There was a not significant tendency for more self-acquired ortheses in the group treated by general practicioners (p = 0,2669).

**Conclusion:**

Patients who first present at the ER with a lateral ankle ligament injury grade I-II can be referred to a general practitioner for follow-up treatment without affecting patient satisfaction regarding treatment and treatment outcome.

## Background

In some Western countries an increasing number of patients seeks help directly at emergency department without seeing their general practitioner first even for minor complaints as inversion trauma of the ankle [1] The occurrence of these acute lateral ankle ligament injuries is estimated at 1:10'000 people per day with a major impact on health care costs [2,3]. This number translates into approximately 650 lateral ankle ligament injuries per day in Switzerland, with a high incidence rate for certain sports [4-6]. In a Scandinavian study acute lateral ankle ligament injuries accounted for 7–10% of all ER admittances, which makes these injuries one of the primary reason for admittance to the emergency room in those countries [7]. Most of the patients are younger than 35 years, with the broadest representation of patients between the age of 15–19 years and an elevated risk especially for young female athletes [4,8,9].

After having eliminated the possibility of a fracture, early functional treatment is the therapy of choice for mild and acute forms of lateral ankle ligament injuries since it there are no surgery related risks as infections, thrombosis, embolism, scars, pain, wound healing problems or anesthesia related adverse effects. The period of recovery has shown to be shorter in non-operative treatment with the same long-term functional results and joint stability [10-17]. From a socio-economic standpoint, this kind of therapy is clearly preferable to other forms of therapy, in particular surgery, but also immobilization [14,15,18]. There is no strong evidence which functional treatment is best suited for these grade I-II lateral ankle ligament injuries [19] Contrary to recommendations of other authors, in our institution we only apply an elastic bandage to prevent swelling in initial phase even for grade III injuries and do not generally advocate the use of stabilizing semi rigid ortheses for the non athletic population [5,18-20].

With regard to bring down costs produced by minor midfoot and ankle injuries our unit already developed the 'Bernese Ankle Rules' which are based on the original 'Ottawa Ankle Rules' and could significantly enhance the specifity for detecting fractures of the ankle and lowering the amount of unnecessarily obtained radiographs [21]. Aside of the initial diagnosis and treatment also follow-up treatment of patients in emergency units requires large amounts of personnel and logistical resources, and is much costlier than treatment by a general practitioner [22,23]. The goal is therefore to refer these patients as quickly as possible to their general practitioner for follow-up treatment. The question that arose was whether the patients who were admitted to the emergency room of the University Hospital with a mild lateral ankle ligament injury reported reduced satisfaction with regards to their therapy as well as the treatment outcome if they are referred to their general practitioner for follow-up care. Objective of this study was to investigate the influence of similar aftercare under different settings (ER vs GP) on patients satisfaction after minor trauma as ankle sprains.

## Methods

100 consecutive patients who were admitted to our trauma center between May and October 2004 with lateral ankle ligament injuries of all grades were included in this prospective randomized study. Patients agreed to participate befor randomization. The protocol was presented to the ethical committee of the University of Bern but no formal approval was necessary as the study was classified as quality control investigation of an established treatment.

Osseous lesions were excluded by clinical examination according to the 'Bernese ankle rules' and by additional antero-posterior and lateral radiographs [21]. The exclusion criteria were: non-obvious supination trauma, previous treatment by a general practitioner or in a different clinic, rupture of deltoid ligament, fractures, and refusal to participate on the part of the patient.

Regardless of the grade of the injury the patients received an early functional therapy, consisting of early antiphlogistic measures according the RICE procedure (Rest – Ice – Compression – Elevation), elastic support bandage, early return to full weight bearing after only a short period of immobilization and instruction for motion exercises to be begun at home after the acute phase. These early active and passive joint movements promote healing and prevent edema [24]. The physical therapy exercises were developed in collaboration with the Swiss Sports Institute (Eidgenössische Turn- und Sportschule in Magglingen, ESSM) and are designed to strengthen the peroneal muscles and improve neuromuscular control through proprioceptive training [16,25]. Most patients were confident with an instructional handout for range of motion, weight bearing and neuromuscular training exercises; only a small percentage (less than 10%) demanded physiotherapeutic guidance. All patients were informed that we do not recommend the use of a semi-rigid ankle ortheses.

Patients then were randomly assigned into two groups: Group ER: emergency room (n = 50, 22 males, 28 female, average age = 34 years); Follow-up within one week in our clinic. Group GP: general practitioner (n = 50, 25 male, 25 female, average age: 35 years); Follow-up within one week at the general practitioner's office. Both groups had a final control in our clinic 2 months after their trauma when the patients were asked to fill a questionnaire for evaluation of subjective satisfaction regarding treatment management and outcome (Table 1). On this protocol there was room left for additional information as the independent acquisition of stabilizing ortheses or the like. At final control the patients underwent a standardized physical examination for objective measure (Table 2). No stress radiographs were performed at any time.

**Table 1:** Questionnaire for evaluation of subjective satisfaction regarding therapy and treatment outcome.

**Satisfaction regarding therapy and treatment outcome**
**Please define your level of satisfaction regarding treatment and therapy:**

very satisfied satisfied dissatisfied disappointed very disappointed

If dissatisfied or disapointed please specify:

**Please define your level of satisfaction regarding treatment outcome:**

very satisfied satisfied dissatisfied disappointed very disappointed

If dissatisfied or disappointed please specify:

**Did you acquire a stabilizing ortheses independently?**

Additional remarks:

**Table 2:** Protocol and results of objective examination at follow up one week (only group ER) and two months after lateral ankle injury (both groups).

		ER 1 wk		ER 2 mts		GP 2 mts	
		
		[no]	[%]	[no]	[%]	[no]	[%]
swelling and/or haematoma	malleolus lateralis	34	70.4	11	22.5	14	29.2
	
	malleolus medialis	2	4.1	2	4.1	2	4.2

pain at palpation of:	capitulum fibulae	0	0	0	0	0	0
	
	malleolus lateralis	8	16.3	0	0	0	0
	
	malleolus medialis	4	8.2	0	0	0	0
	
	lig talofibulare anterius	35	71.4	20	40.1	18	37.5
	
	metatarsalia	1	2	0	0	0	0
	
	anterior syndesmosis	14	28.6	1	2	0	0

[number of patients/percentage]

Statistical analysis then was performed to evaluate the dependence of patients' satisfaction on where the follow-up was carried out by using a Chi-squared and Fishers' exact test with a significance level set at p < 0,05.

## Results

The final check-up for all patients took place 2 months after the trauma (n = 97, 2 patients died (1 suicide/1 of natural causes), one patient moved away without leaving a forwarding address).

**Figure 1:**
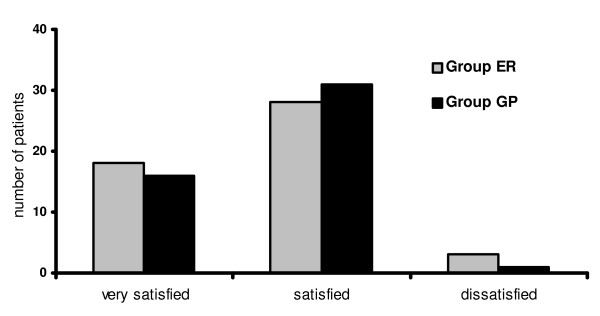
**Subjective satisfaction with regard to treatment of patients 2 months after acute lateral ligament injury**. Aftercare conducted by emergency room physicians (group ER) or general practitioners (group GP).

Satisfaction rate regarding therapy two months after the trauma was high for both groups: in the ER group, 18 patients (37%) were very satisfied, 28 patients (57%) were satisfied, three patients (6%) were dissatisfied. In the GP group, 16 patients (33%) were very satisfied, 31 patients (65%) were satisfied and one patient (2%) was dissatisfied (fig. 1). The main reason for dissatisfaction was a feeling of being under-treated. The differences between the two groups are not significant (p = 0.7406). In both groups no patients were found to be disappointed or very disappointed.

**Figure 2:**
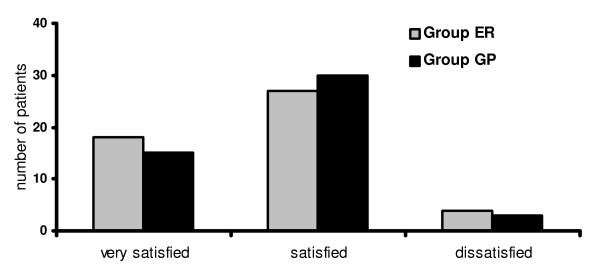
**Subjective satisfaction with regard treatment outcome of patients 2 months after acute lateral ligament injury that received an early functional treatment**. Aftercare conducted by emergency room physicians (group ER) or general practitioners (group GP).

Satisfaction regarding treatment outcome was evaluated two months after the trauma: In the ER group, 18 patients (37%) were very satisfied, 27 patients (55%) were satisfied and 4 patients (8%) were dissatisfied. The main reason for dissatisfaction was persistent swelling and pain. In the GP group, 15 patients (31%) were very satisfied, 30 patients (63%) were satisfied and 3 patients (6%) were dissatisfied. No patients were disappointed or very disappointed. The uniform reason for dissatisfaction in this group was persistent pain (fig. 2). Again the differences between the two groups were not significant (p = 0.7631).

36 out of a total of 97 patients (15 in ER group/21 in GP group) acquired an ankle brace (mostly Aircast^® ^(Summit, NJ USA) or Malleoloc^® ^(Bauerfeind AG, Zeulenroda, Germany)), i.e. 39% of all patients. There was a tendency for more self-acquired ortheses in the GP group although there was no significant correlation to where the treatment took place or the level of satisfaction (p = 0.129). 6 patients in the ER group and 3 patients in the GP group required physiotherapy (p = 0.253) (Table 3).

**Table 3:** Overview of number/percentage of patients who needed physiotherapeutic assistance or bought an ankle ortheses independently.

	Ankle brace	Physiotherapy
Group ER (n = 49)	15 (30.6%)	6 (12.2%)

Group GP (n = 48)	21 (43,8%)	3 (6.3%)

**Figure 3:**
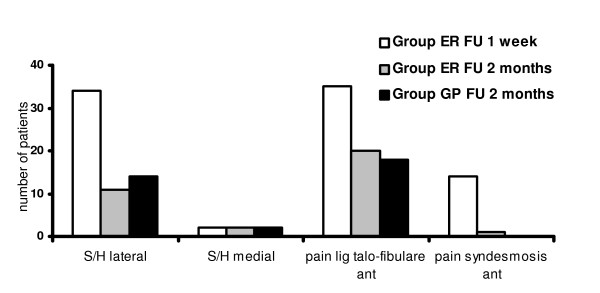
**Objective measure of treatment outcome one week (group ER) and two months (both groups) after acute lateral ligament injury**. Aftercare conducted by emergency room physicians (group ER) or general practitioners (group GP) [S/H = swelling and/or haematoma.

At 2 months after trauma at the objective physical examination swelling and/or haematoma above the lateral malleolus was present in 11 patients (22%) in the ER group and 14 patients (29%) in the GP group. Swelling and/or haematoma above the medial malleolus were diagnosed in 2 patients in each group (4% in each group). Pain with pressure above talofibular ligamentswas only diagnosed in 20 patients (40%) in the ER group and in 18 patients (38%) in the GP group. In the ER group, 1 patient (2%) complained of persistent pain with pressure in the area of the syndesmosis. Pain with pressure was not reported for any of the other pressure areas (table 2, fig. 3).

Objective collection of data could again not find significant differences between the two groups.

## Discussion

At our trauma center, acute lateral ankle ligament injuries of all grades are addressed with early functional treatment. The initial treatment is based on the RICE method (Rest – Ice – Compression – Elevation) in conjunction with the use of crutches, followed by physical therapy exercises which the patient can do independently at home. These early active and passive joint movements prevent edema, are designed to strengthen the peroneal muscles and improve neuromuscular control through proprioceptive training [16,24-26].

The analgesics that are prescribed are limited to paracetamol, mefenamic acid and/or Cox-2 inhibitors. Taking non-steroidal anti-inflammatory drugs has no effect on the healing process [24].

The basis of our study was the question whether patients, who chose the emergency room of the University Hospital for initial diagnosis, are satisfied with our initial therapy, and whether there is a difference in the level of satisfaction between the patients who receive follow-up care at our clinic or at their general practitioner's office. Since our clinic does not prescribe ankle braces (Malleoloc or Aircast), the additional question arose as to whether the patients acquired such stabilizing ortheses themselves or were prescribed ankle braces by their general practitioner during the follow-up visit.

The results confirm that the method of follow-up care has neither influence on either patient satisfaction nor the subjective or objective treatment outcome. It is encouraging that the results show that patients are satisfied with the early functional treatment and don't feel as if they were under-treated. Follow-up care can be transferred to general practitioners without affecting patient satisfaction. This leads to a significant decrease in the costs for follow-up care. These results are consistent with other investigations that report a high satisfaction rates at lower costs for patients with minor injuries when treated by their chosen family physician [22,23,27].

The fact that 39% of all patients independently acquired stabilizing orthoses such as Aircast, Malleoloc etc. presents the challenge of patient education for the doctor making the initial diagnosis. Although there was a slight tendency for more self-acquired ortheses in the second group (GP) there was no significant difference. Use of stabilizing orthoses has no influence on the treatment outcome at two months follow-up, though the patient's comfort level may be improved through external stabilization and studies report a faster return to work [19]. However, this increased comfort level comes with a high price tag: with an incidence rate of 1 trauma/10.000/day [2,3],. the average number of acute lateral ankle ligament injury cases per year in Switzerland would be approx. 240'000. 39% of the patients, i.e. 93'000 patients per year, acquired stabilizing ortheses, at an average cost of CHF 100, which amounts to a total amount of additional costs of approx. CHF 9.3 million per year.

The reasons for acquiring auxiliary devices are varied. The two most common answers given were increased comfort and the fact that friends/family members were treated with stabilizing ortheses.

## Conclusion

Our patients with lateral ankle injuries receiving early functional treatment report a high satisfaction rate (Additional file 1). Patient satisfaction does not depend on where follow-up care is performed (emergency room vs. general practitioner). Subsequently, patients can be referred to their general practitioner for follow-up care. This can ease the strain on resources at the emergency units of hospitals and leads to significant cost savings and continous physician-patient relationship.

Independent acquisition of orthopedic auxiliary devices (Aircast, Malleoloc etc.) is not significantly different between the two groups, yet amounts to the considerable number of 39% and additional costs.

## Competing interests

The authors declare that they have no competing interests.

## Authors' contributions

PS did the clinical trial and participated in the design of the study and the statistical analysis. LB paticipated in the design of the study and controlled the use of the bernese ankle rules. SE patricipated in the design of the study. HZ did the one part of the statistical analysis. AE participated in the design of the study and performed the other statistics. All Authors read and approved the final manuscript.

## Pre-publication history

The pre-publication history for this paper can be accessed here:



## Supplementary Material

Additional File 1 **Flowchart.** Outcome of lateral ankle injuries. Click here for file
