# Supplementary figures and images for: Outcome and patients' satisfaction after functional treatment of acute lateral ankle injuries at emergency departments versus family doctor offices
Source: BMC Fam Pract. 2008 Dec 23;9:69. doi: 10.1186/1471-2296-9-69 (PMC2631016; doi:10.1186/1471-2296-9-69)

## Slide 1
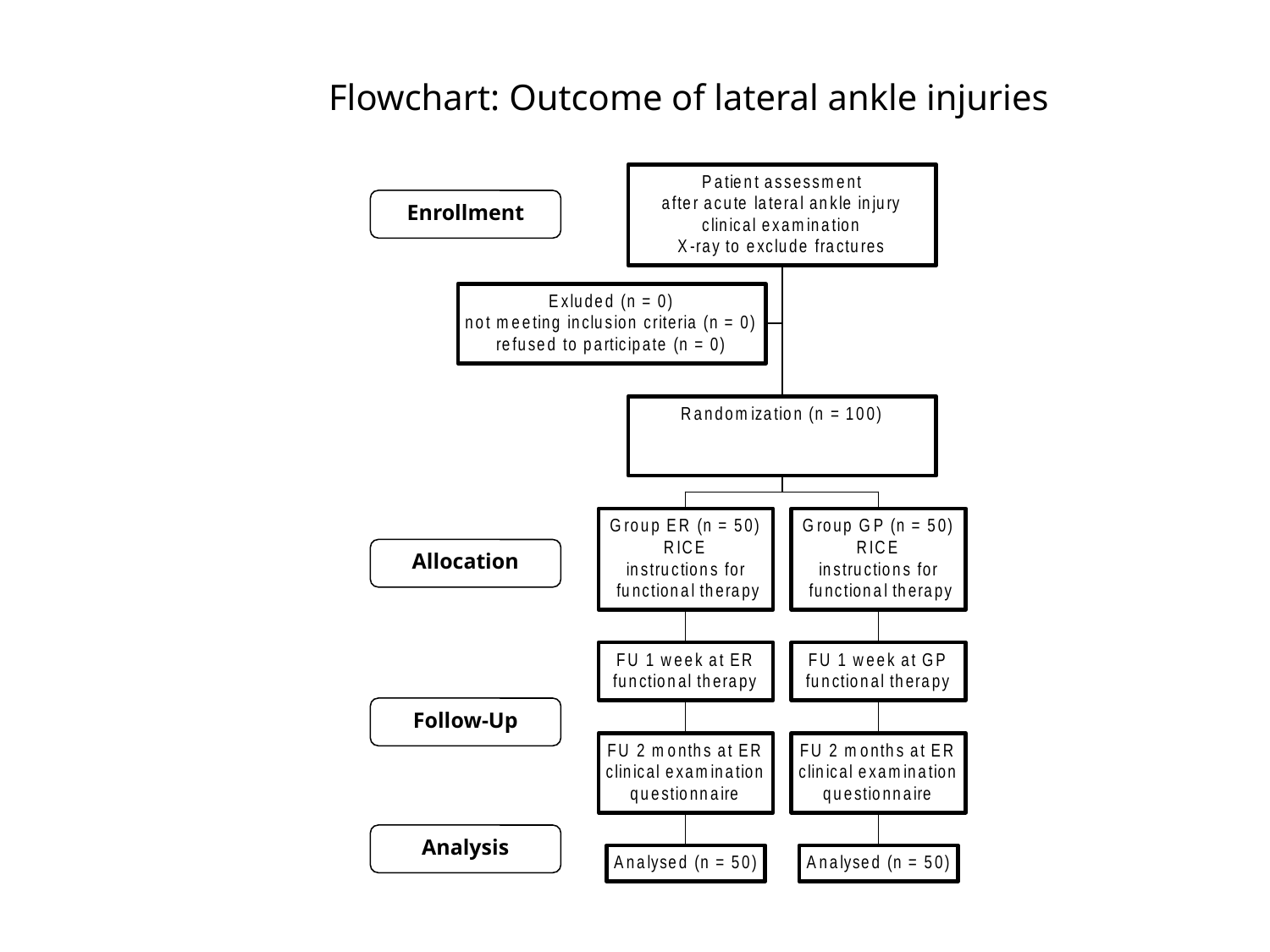

# Flowchart: Outcome of lateral ankle injuries
Enrollment
Allocation
Follow-Up
Analysis

Supplement: Additional File 1 — Flowchart. Outcome of lateral ankle injuries. [file 1471-2296-9-69-S1.ppt]
